# Data resource profile: the Korea National Hospital Discharge In-depth Injury Survey

**DOI:** 10.4178/epih.e2021052

**Published:** 2021-08-17

**Authors:** Yeon-Kyeng Lee, Sung Ok Hong, Soo-Jung Park, Mijin Park, Kyunghae Wang, Mini Jo, Jeongah Oh, Sin Ae Lee, Hyeon Ju Lee, Jungeun Oh, Dosang Lim, Sanghui Kweon, Youngtaek Kim

**Affiliations:** 1 Korea Disease Control and Prevention Agency, Cheongju, Korea; 2 Public Health and Medical Service Office, Chungnam National University Hospital, Daejeon, Korea

**Keywords:** Hospital records, Inpatients, Injury surveillance, Epidemiology

## Abstract

The Korea National Hospital Discharge In-depth Injury Survey (KNHDIS), which was started in 2005, is a national probability survey of general hospitals in Korea with 100 or more beds conducted by the Korea Disease Control and Prevention Agency (KDCA). The KNHDIS captures approximately 9% of discharged cases from sampled hospitals using a 2-stage stratified cluster sampling scheme, among which 13% are injury related cases, defined as S00-T98 (injury, poisoning, and certain other consequences of external causes) using International Classification of Diseases, 10th revision codes. The KNHDIS collects information on characteristics of injury-related discharges in order to understand the scale of injuries, identify risk factors, and provide data supporting prevention policies and intervention strategies. The types of data captured include the hospitals’ information, detailed clinical information, and injury-related codes such as the mechanism, activities undertaken when injured (sports, leisure activities, work, treatment, and education), external causes of the injury, and location of the occurrence of the injury based on the International Classification of External Causes of Injuries. Furthermore, the means of transportation, risk factors for suicide, and toxic substances are recoreded. Annual reports of the KNHDIS are publicly accessible to browse via the KDCA website (http://www.kdca.go.kr) and microdata are available free of charge upon request via email (kcdcinjury@korea.kr).

## INTRODUCTION

Injuries constitute a major public health problem, killing more than 5 million people worldwide each year (9% of annual deaths) and causing many more cases of disability. In Korea, the death rate from injuries dropped from 61.2 deaths per 100,000 people in 2009 to 56.5 deaths per 100,000 people in 2019, with traffic accidents comprising approximately 10% of deaths among those aged 1 year to 29 years old in 2019 [1]. Injuries, both unintentional and intentional, are considered to be largely preventable events. Furthermore, injuries and their burden can be reduced by implementing effective measures for prevention and treatment [2].

In 2001, the World Health Organization (WHO) recommended implementing an injury surveillance system to educate individuals about injury prevention and treatment and improve recovery outcomes. Many countries, including the United States, Canada, Australia, and China, implemented the WHO’s injury surveillance guidelines to assess patterns and trends related to injuries [3]. In 2005, the Korea Disease Control and Prevention Agency (KDCA) launched an injury surveillance system called the Korea National Hospital Discharge In-depth Injury Survey (KNHDIS) that uses hospital-based data, patient demographic data (age, sex, and geographic area), details of patients’ injuries (intent, place, mechanism, and nature of injury), and contextual data (date of admission and discharge, outcomes, payment information) to gain a better understanding of the scale of the effects of injuries nationwide, identify risk factors, and provide data that support prevention policies and intervention strategies suggested in the WHO injury surveillance guidelines. The KNHDIS is an integrated system of data collection, analysis, interpretation, and communication. It is a national probability survey that has been conducted annually since its inception.

The KNHDIS defines patients with injury-related discharges as S00-T98 (injury, poisoning, and certain other consequences of external causes) according to the International Classification of Diseases, 10th revision (ICD-10) [4], which is designated in addition to the main diagnosis or sub-diagnosis code.

## DATA RESOURCE AREA AND POPULATION COVERAGE

The KNHDIS is an ongoing probability survey that targets injured patients admitted to hospitals. The scope of the KNHDIS encompasses patients discharged from all acute general hospitals with 100 or more beds, excluding single specialized hospitals, nursing hospitals, geriatric hospitals, veterans’ hospitals, military hospitals, and rehabilitation hospitals. In addition, cases in which patients only stayed in the emergency room or had normal full-term spontaneous delivery are excluded [5]. In the past, KNHDIS data have captured multiple admissions without distinguishing between the initial admission and subsequent readmissions, except for the first year of the survey, during which multiple events were excluded altogether.

### Data collection procedures

Two data collection procedures are used for the KNHDIS. One is an automated system in which the sampled hospitals utilize the automated system use an electronic medical record system to automatically extract the vaiables required for the Korea Uniform Hospital Discharge DataSet (KUHDDS), after which the hospital’s staff conduct surveys for the KUHDDS variables and injuryrelated codes. The KUHDDS included patients’ information on demographic, admission and medical categories. Then, hospitals transmit the data electronically to the data system operated by the KDCA, known as the integrated disease health management system (the IS, http://is.kdca.go.kr) [6].

The second procedure is a manual system in which sample selection and transcription of information from hospital records to abstract forms on the IS are performed by trained research staff at the KDCA.

Approximately 68.6% of sampled hospitals provide their data through the automated system, and hospitals that use the automated system accounted for 89.0% of the total data in 2018. As of 2019, the survey participation rate of sampled hospitals tended to slightly decrease over time—from 98.0% in 2004 to 95.5% in 2018 (Table 1) [7].

### Sampling design

The original sample in 2005 was selected from a pool of general hospitals listed in the national patient survey. A 2-stage stratified cluster sampling scheme was applied, and the hospitals were stratified into 4 strata based on the number of beds (100-299, 300-499, 500-999, and ≥ 1,000) and geographic location (at city and provincial levels, referred to as “*si*” and “*do*,” respectively). Sixteen regional emergency medical centers, 4 specialized emergency medical centers, and 5 specialized burn care hospitals were mandatorily selected in 2007 [8]. In the first stage of the scheme, individual hospitals are selected as the primary sampling unit (PSU). In the second stage, discharged cases from the sampled hospitals are selected as the secondary sampling unit (SSU). The selection probabilities of the sample plan are updated periodically based on previous survey results and variance in the number of hospitals. The sample size was 150 hospitals in the first year of the survey and had been expanded to 200 hospitals by 2017 [9]. The selected discharge cases comprise approximately 9% of the total discharge cases each year (approximately 10% until 2006), but they did not exceed 6,000 cases per hospital. The selection process is performed using the systematic sampling method. The number of cases selected from hospitals with non-automated KUHDDS is based on the number of beds according to the following system: a maximum of 300 cases are selected from hospitals with 100-199 beds, 420 cases from hospitals with 200-299 beds, and 540 cases for hospitals with more than 300 beds. For the 2010 survey population, the probability was doubled for children aged 0 to 4 and female aged 25 to 35 due to high estimation errors in the previous survey results. In the future, the sample size will be increased to as many as 250 hospitals to better estimate the representative statistics of each region.

The KNHDIS data capture multiple admissions without distinguishing between the initial admission and the subsequent readmissions, except in the first surveyed year, which excluded multiple events. Injury cases accounted for approximately 13% to 15% of total discharges each year, including multiple events. In 2018, 302,593 hospitalizations were reviewed, from which 38,109 injury cases were identified and examined (Table 2).

### Estimation of total discharged cases

The survey is a complex sample survey, and appropriate weights are applied for the estimation. The number of injury cases is estimated using the estimation of the mid-year population for each year. Linear variance estimation is carried out using the Taylor series and the SURVEYMEANS procedure (SAS Institute Inc., Cary, NC, USA), using the latest version available at the time of the survey (version 9.4 was the most recent version used in 2019).

The weighting is calculated by multiplying the probability of the hospital selection (PSU) by the probability of the patient selection (SSU). The formula for estimating the total ($\hat{Y}$) is defined as follows:


$$\hat{Y}=\sum_{h=1}^{H}\frac{N_{h}}{n_{h}}\sum_{i=1}^{n_{h}}\frac{M_{hi}}{m_{hi}}\sum_{j=1}^{m_{hi}}y_{hij}=\sum_{h=1}^{H}\sum_{i=1}^{n_{h}}\sum_{j=1}^{m_{hi}}w_{hij}\cdot y_{hij}$$

where,

*h*: stratum h by hospital size (*h*= 1,2, *H*= 4)

*i*: *i^th^* hospital in stratum *h, i*= 1, 2,…, *n_hi_*

*j*: *j^th^* patient of *i^th^* hospital in stratum *h, j= 1,2, … m_hi_*

*N_h_* is the number of target hospitals in stratum *h*.

*n_h_* is the number of hospitals to be sampled in stratum *h*.

*M_hi_* is the total number of discharged patients from the *i^th^* hospital in stratum *h*.

*m_hi_* is the number of sampled discharged patients from the *i^th^* hospital in stratum *h*.

*W_hij_* is the weighting of discharged patients to be sampled from hospital j in stratum *h*.


$$w_{hij}=\frac{N_{h}}{n_{h}}\times \frac{M_{hi}}{m_{hi}}$$

The variance of the estimated total number of discharged patients $\hat{Y}$ is computed using the following formula:


$$\hat{V}(\hat{Y})=\sum_{h=1}^{H}\hat{V_{h}}(\hat{Y})$$

where,


$$\hat{V_{h}}\left(\hat{Y}\right)=\frac{n_{h}}{n_{h}-1}\sum_{i=1}^{n_{h}}(y_{hi\cdot }-y_{h\cdot \cdot }^{-}{)}^{2},y_{hi\cdot }=\sum_{j=1}^{m_{hi}}w_{hij}\cdot y_{hij},y_{h\cdot \cdot }^{-}=(\sum_{i=1}^{n_{h}}y_{hi\cdot })/n_{h}$$

The standard error is computed as the square root of the total variance:


$$\sqrt{\hat{V}(\hat{Y})}.$$

### Data validation and quality

The KDCA developed a quality management program that involves first checking the data transmitted into the IS. Data are subject to automatic quality control checks upon submission of the original entry. Various errors—including omission of essential items, duplicate transmissions of the same data, as well as errors in admission and discharge dates, code values for each item, operation dates, and injury occurrence dates—are analyzed and returned. During the second step, a specialized quality control team within the KDCA verifies the items surveyed and identifies any logical errors that may exist in the items (e.g., discrepancies between patients’ ages and the sources of medical expenses; external injury codes and information gathered from the in-depth survey; age, location, and activity leading to injury; age, diagnosis, and surgical code; and sex-specific diagnoses and surgical codes). Third, external experts review the cases of patients who had been hospitalized for more than 180 days, which are then excluded from the nationally representative dataset. Information related to those patients’ diseases and treatments, such as codes for diagnoses, procedures, and the original causes of injuries in patients, as well as information related to deceased neonatal patients, is provided as raw data. Any errors or information requiring reconfirmation are corrected with the hospital.

## DATA COLLECTION

### Patient and clinical information

The data collected include hospital information; patients’ demographic information including age, sex, zip code of residence, primary payer/insurance status; detailed clinical information; and injury-related codes related to the mechanism and location of the injury based on the International Classification of External Causes of Injuries, version 1.2 (ICECI) [10], with 30 variables collected in the core dataset (Table 3).

Primary diagnoses and additional diagnoses are coded according to the ICD-10. Procedures were coded according to the ICD10 and the International Classification of Diseases, 9th revision, Clinical Modification (ICD-9-CM). The ICD-10 was used from 2005 to 2014, and the Korean Standard Classification of Diseases7th edition (KCD-7) [11] by Statistics Korea was used from 2015 onwards. The KCD-7 is the Korean version of the ICD-10 and includes Korea-specific disease codes. Primary procedures that are defined as surgical operations and additional procedures include endoscopic polypectomy, Gamma Knife radiosurgery, extracorporeal shock wave lithotripsy, and special examinations for diagnosis purposes. For each discharge record, up to 20 additional diagnostic and additional procedure codes were collected beginning with the second KNHDIS. Apart from data on diagnoses and procedures, the survey contains information on the dates of admission, each coded procedure, and discharge and injury occurrence as well as on primary payer/insurance status such as if patients were covered by national health insurance, Medical Aid (Medicaid, Medicare), car insurance, or industrial accident compensation insurance, or if they were uninsured.

### Injury information

The survey’s injury-related content was developed based on the ICECI, as recommended by the WHO. Key data elements on injuries include the intent of injury, place of occurrence, injury mechanism, and activities undertaken when injured (sports, leisure activities, work, treatment, or education). Discharges with diagnoses coded as S00-T75 and T79 require further information collection on the external causes of injuries (V-Y code) and all 10 variables in the injury information dataset (Table 3). Up to 2 codes are collected to classify external causes of injury to understand the outcomes after the original injury. Injuries are classified according to intention using 4 categories: unintentional injuries, intentional injuries, violence-related injuries, and legal intervention. Possible locations where injuries can occur are classified as residences, schools or school areas, commercial facilities, farms, medical facilities, or cultural facilities such as amusement parks, public buildings, or industrial/construction sites and are linked to the external cause of injury. The injury mechanism pertains to information about the method or instrument that caused the injury. The outcome of the injury patient at the time of discharge is evaluated with medical records and coded using the Glasgow Outcome Scale of 1 to 5 (1 for dead, 2 for persistent vegetative state, 3 for severe disability, 4 for moderate disability, and 5 for good recovery).

The following items are assessed on an as-needed basis: the means of transportation (pedestrian, bicycle, motorcycle, car, bus, or airplane), suicide risk factors (conflict with family members, disease, financial problems, death in the family, or abuse), and exposure to toxic substances. For cases with the codes T78, T80-T98, and Y40- Y98, only external causes of injuries are surveyed (E, V code according to the ICD-9 and V, W, X code according to the ICD-10). The nature of injuries was surveyed separately until 2005, after which it was changed to be generated based on the S00-T98 code of the primary diagnosis or additional diagnosis. The injury discharge rate of each surveyed year was estimated using the estimated population of that year according to demographic characteristics, intent, injury mechanism, etc., from 2004 to 2017 (Table 4).

### Data resource use

The KNHDIS provides a unique data source not currently available elsewhere in Korea and has been widely used by various academic researchers and policymakers. Annual reports have been published in the second half of the following year since 2006 (target data of 2004) [12]. The KNHDIS provided health statistics for the development of injury prevention-related objectives for the Health Plan 2020, which is the national comprehensive health plan devised by the Korean Ministry of Health and Welfare [13]. Specifically, the KNHDIS provided the rates of admission to hospitals for causes related to intentional or unintentional injuries and traffic accidents. In support of the presidential agenda goals, the KNHDIS has also provided statistics on 7 indicators related to safetyrelated objectives for children under the age of 14 since 2011 [14]. These included rates of admission caused by intentional/unintentional injuries, poisoning, sports/recreation injuries, traffic accidents, and pedestrian traffic accidents. The data was also used to revise the KCD by Statistics Korea [15]. With over 100 publications as of July 2020, the KNHDIS has been used to answer a variety of relevant research questions. The main research issues it addresses include the effects of disease burden [16-18], the risk factors for hospitalization duration [18-21], and epidemiologic characteristics of injuries [8,22-24]. A full list of publications that use KNHDIS data in domestic and international journals is available from the supplementary data section of the *International Journal of Epidemiology* website. The annual KNHDIS symposium held by the KDCA has been conducted 14 times as of 2019, and is attended by approximately 250 researchers each year. Comprehensive injury fact sheets have been published annually since 2011 in collaboration with the KDCA and various other agencies, including the fire agency, the statistics agency, the Rural Development Administration, the National Medical Center, the National Health Insurance Corporation, the Road Traffic Authority, and the School Safety and Insurance Federation. Furthermore, KNHDIS data have increased public awareness regarding the importance of injury prevention, with a chapter about injury prevention having been recently added to Korean language textbooks for fourth-graders.

### Ethics statement

The study was exempt from institutional review board approval as the KNHIDS was conducted a part of national injury surveillance system and all analyses in this study were used public-open data.

## STRENGTHS AND WEAKNESSES

The KNHDIS is an ongoing survey that reflects a nationally representative sample in Korea. It is a valuable source for understanding injury-related information and has been used to improve policies related to injury prevention. It also enables the identification and analysis of the trend of injuries in a time-sequential manner and continually provides systematic national health and medical statistics related to injuries for the public and the academic sector.

The KNHDIS covers not only patients with health insurance provided by the Korea National Health Insurance Service but also those who are covered by other types of insurance, such as industrial accident insurance and car insurance. Furthermore, the contents of the survey are coded using the ICD-10 and ICECI, which makes international comparisons possible.

The KNHDIS has several limitations. The KNHDIS only surveys hospitals with more than 100 beds. As such, additional surveys on hospitals with fewer than 100 beds are needed, as well as surveys on outpatient injuries. In addition, the dataset does not distinguish between cases of readmission to multiple hospitals since it does not collect personal linkable identification codes. In other words, all of the data related to 1 particular discharge case are from 1 hospital only.

## DATA ACCESSIBILITY

Data are available for all years during which the survey was conducted. The annual reports can be downloaded at the KDCA website (http://www.kdca.go.kr) and the Korea Statistics website (http://mdis.kostat.go.kr). Microdata are available upon request by email (kcdcinjury@korea.kr) via an application (https://www.kdca.go.kr/board/board.es?mid=a20507030000&bid=0020). Qualified applicants can submit an application form summarizing their proposed research projects for which they plan to use the KNHDIS. Data will be transferred to successful applicants by the KDCA injury.

## Figures and Tables

**Table 1.** The number of sampled hospitals, their rates of participation, and the number of surveyed cases in the KNHDIS

Target year	No. of target population hospitals					No. of sample hospitals (A)					No. of participating hospitals (B) (A/B, %)				
	Sum	100-299	300-499	500-999	≥1,000	Sum	100-299	300-499	500-999	≥1,000	Sum	100-299	300-499	500-999	≥1,000
2004	512	334	84	84	10	150	70	27	43	10	147 (98.0)	67 (95.7)	27 (100)	43 (100)	10 (100)
2005	512	334	84	84	10	150	70	27	43	10	147 (98.0)	68 (97.1)	27 (100)	42 (97.7)	10 (100)
2006	512	334	84	84	10	150	70	27	43	10	144 (96.0)	65 (92.9)	26 (96.3)	43 (100)	10 (100)
2007	561	408	75	71	7	170	102	19	42	7	165 (97.1)	100 (98.0)	18 (94.7)	40 (95.2)	7 (100)
2008	561	408	75	71	7	170	102	19	42	7	170 (100)	102 (100)	19 (100)	42 (100)	7 (100)
2009	561	408	75	71	7	170	102	19	42	7	170 (100)	102 (100)	19 (100)	42 (100)	7 (100)
2010	561	408	75	69	9	170	103	18	40	9	166 (97.6)	101 (98.1)	18 (100)	39 (97.5)	8 (88.9)
2011	561	408	75	69	9	170	103	17	41	9	166 (97.6)	101 (98.1)	17 (100)	41 (100)	7 (77.8)
2012	561	408	75	69	9	170	103	17	41	9	166 (97.6)	101 (98.1)	17 (100)	40 (97.6)	8 (88.9)
2013	561	408	75	69	9	170	103	17	41	9	164 (96.5)	99 (96.1)	17 (100)	41 (100)	7 (77.8)
2014	561	408	75	69	9	170	103	17	41	9	164 (96.5)	98 (95.1)	17 (100)	41 (100)	8 (88.9)
2015	561	408	75	69	9	170	102	18	41	9	165 (97.1)	99 (97.1)	18 (100)	40 (97.6)	8 (88.9)
2016	561	408	75	69	9	170	101	18	42	9	161 (94.7)	95 (94.1)	17 (94.4)	41 (97.6)	8 (88.9)
2017	572	414	64	77	17	200	95	31	57	17	188 (94.0)	91 (95.8)	28 (90.3)	55 (96.5)	14 (82.4)
2018	572	414	64	77	17	200	95	31	57	17	191 (95.5)	91 (95.8)	30 (96.8)	55 (96.5)	15 (88.2)

KNHDIS, Korea National Hospital Discharge In-depth Injury Survey.

**Table 2.** KNHDIS databases between 2004 and 2008

Target year	No. of participating hospitals	No. of discharged cases	No. of injury-related cases
2004	147	175,100	26,791
2005	147	161,997	24,818
2006	144	170,008	25,511
2007	169	179,094	27,291
2008	170	190,074	28,031
2009	170	198,586	29,030
2010	166	220,838	31,654
2011	166	225,105	31,425
2012	167	235,526	32,758
2013	163	214,605	30,589
2014	165	224,061	31,389
2015	165	227,615	31,784
2016	162	235,579	30,829
2017	188	291,786	37,752
2018	191	302,593	38,109

KNHDIS, Korea National Hospital Discharge In-depth Injury Survey.

**Table 3.** The KNHDIS data variables elements (as of June 2020)

Subjects			Contents
Patients			
	Hospital information		Hospital identification code, no. of beds in the hospital, hospital address
	The KUHDDS		
		Demographics	Gender sex, age at admission, date of birth, zip code
		Admission information	Expected source of payments, admission date, discharge date, admission route (e.g., emergency, outpatient)
		Medical information	Primary diagnosis KCD-10 (KCD-7) code, additional diagnosis KCD-10 (KCD-7) code (up to 20)^1^, external cause of injury (up to 2)^2^, primary procedure ICD-9-CM, date of procedure, additional procedure code (up to 20)^1^, discharge method, underlying cause of death, discharge disposition (e.g., home, other health care facility, expired), treatment result (e.g., improved, deceased)
Injured patients (injury information)			
	S00-T75, T79 (ICD-10)		Intent, place of occurrence, activity when injured, mechanism of injury, date of injury occurrence, nature of injury (e.g., superficial injury, open wound, fracture; generated from ICD code T00-07, T20-632), mode of transport, proximal risk factors for intentional self-harm (e.g., conflict with family members, physical illness, financial problems), exposure to toxic substances, Glasgow Outcome Scale score, external causes of injuries (V-Y code)
	T78, T80-T98, Y40-Y98 (ICD-10)		External causes of injuries (V-Y code)

KNHDIS, Korea National Hospital Discharge In-depth Injury Survey; KUHDDS: Korea Uniformed Hospital Discharge DataSet; KCD, Korean Standard Classification of Diseases; ICD-9-CM, International Classification of Diseases, 9th revision, Clinical Modification.

1 Surveyed up to 5 in 2004.

2 Surveyed up to 1 in 2004.

**Table 4.** Estimated injury discharge rates^1^, 2004-2017

Variables		2004	2005	2006	2007	2008	2009	2010	2011	2012	2013	2014	2015	2016	2017
Total		9,081	9,477	9,490	10,091	10,518	11,131	11,979	12,488	13,093	13,184	13,505	13,800	14,218	14,199
	Non-injuries	7,722	8,007	7,995	8,551	8,891	9,464	10,136	10,663	11,164	11,222	11,499	11,732	12,162	12,042
	Injuries	1,359	1,470	1,495	1,541	1,628	1,667	1,843	1,825	1,929	1,962	2,006	2,068	2,056	2,157
Intent		1,627	1,713	1,575	1,787	1,837	1,872	2,057	2,019	2,119	2,118	2,121	2,169	2,087	2,160
	Non-intentional	1,505	159	1,622	1,669	1,721	1,762	1,947	1,913	2,021	2,006	2,015	2,070	2,007	2,082
	Intentional	111	113	115	105	111	106	105	101	91	92	90	82	74	71
	Unknown	10	10	15	12	3	4	5	5	6	20	16	17	6	5
Age (yr)															
	0-14	880	868	835	862	912	872	918	918	963	921	964	901	804	795
	15-24	1,373	1,468	1,495	1,574	1,560	1,573	1,655	1,634	1,697	1,608	1,672	1,747	1,623	1,567
	25-34	1,620	1,697	1,741	1,833	1,863	1,844	1,959	1,929	1,911	1,836	1,811	1,745	1,656	1,639
	35-44	1,824	1,875	1,970	1,900	1,880	1,939	2,004	1,903	1,938	1,947	1,805	1,817	1,743	1,685
	45-54	2,111	2,280	2,315	2,335	2,333	2,463	2,648	2,588	2,626	2,632	2,525	2,564	2,313	2,326
	55-64	2,570	2,705	2,666	2,643	2,613	2,755	2,904	2,894	3,083	3,070	3,044	3,137	3,064	3,263
	65-74	3,072	3,174	34,378	3,201	3,434	3,446	3,967	3,686	3,941	3,941	4,000	3,830	3,896	4,094
	≥75	3,958	4,188	4,451	4,226	4,641	4,481	5,242	5,057	5,644	5,485	5,786	6,101	6,272	6,666
Injury mechanism															
	Traffic accident	669	690	714	746	743	745	772	734	771	729	731	738	670	660
	Fall	463	491	518	504	532	558	654	668	703	748	736	783	792	873
	Struck by/against	164	173	246	243	242	239	268	263	274	248	251	252	231	256
	Stabbing	86	141	87	72	77	68	74	67	69	75	62	68	77	81
	Poisoning	47	47	61	52	55	58	58	55	57	57	59	53	50	45
	Fire/Flame^2^	35	323	35	46	54	61	59	52	56	56	67	58	51	31
	Other	163	152	90	124	134	143	172	180	189	205	215	217	216	214

1 Discharge rate: the discharge rate per 100,000 people used population estimates from the surveyed years.

2 Estimates with a relative standard error of less than 5.
